# Acoustic Droplet Vaporization in Biology and Medicine

**DOI:** 10.1155/2013/404361

**Published:** 2013-11-20

**Authors:** Chung-Yin Lin, William G. Pitt

**Affiliations:** ^1^Department of Chemical Engineering, Brigham Young University, Provo, UT 84602, USA; ^2^Department of Neurosurgery, Chang Gung Memorial Hospital, Taoyuan 333, Taiwan; ^3^Division of Clinical Toxicology, Chang Gung Memorial Hospital, Taoyuan 333, Taiwan

## Abstract

This paper reviews the literature regarding the use of acoustic droplet vaporization (ADV) in clinical applications of imaging, embolic therapy, and therapeutic delivery. ADV is a physical process in which the pressure waves of ultrasound induce a phase transition that causes superheated liquid nanodroplets to form gas bubbles. The bubbles provide ultrasonic imaging contrast and other functions. ADV of perfluoropentane was used extensively in imaging for preclinical trials in the 1990s, but its use declined rapidly with the advent of other imaging agents. In the last decade, ADV was proposed and explored for embolic occlusion therapy, drug delivery, aberration correction, and high intensity focused ultrasound (HIFU) sensitization. Vessel occlusion via ADV has been explored in rodents and dogs and may be approaching clinical use. ADV for drug delivery is still in preclinical stages with initial applications to treat tumors in mice. Other techniques are still in preclinical studies but have potential for clinical use in specialty applications. Overall, ADV has a bright future in clinical application because the small size of nanodroplets greatly reduces the rate of clearance compared to larger contrast agent bubbles and yet provides the advantages of ultrasonographic contrast, acoustic cavitation, and nontoxicity of conventional perfluorocarbon contrast agent bubbles.

## 1. Introduction

Acoustic droplet vaporization (ADV) is a relatively recently exploited phenomenon in which a liquid droplet is induced to form a vapor phase as a result of the application of cyclic pressure waveforms (acoustic waves). While this phenomenon has been described in the literature since 1995 as an imaging application [1–3], it acquired the name “acoustic droplet vaporization” in 2000 [4] along with other proposed and demonstrated applications in gas embolism, thrombolysis, and drug delivery. While ADV has many applications in biology, physics, and engineering, this review will center on its application in clinical medicine, with emphasis on imaging and gas embolization, and on the delivery of therapeutics and markers.

Acoustic droplet vaporization has other synonyms in the literature, such as “phase-shift emulsion” and “ultrasonic droplet vaporization.” The enthusiasm for ADV and its associated processes comes from its useful and unique applications in some of the more challenging issues of medical imaging and therapeutic delivery. At the same time, increasing access to ultrasonic transducers has made ADV more available to researchers in academia and in the clinic.

In theory, ADV could be employed with any liquid that has a normal boiling point near or below body temperature. The physics are based on the vapor pressure of the liquid, which is a function of temperature, and not necessarily based upon the liquid chemistry. However, for medical applications it is essential to use nontoxic biocompatible liquids that are immiscible with water. Fluorocarbons are good candidates, particularly the perfluorocarbons (PFCs) because they have low solubility in aqueous formulations, and they have relatively low toxicity.  Table 1 lists some parameters of candidate alkane perfluorocarbons [5]. Of these, perfluoropentane (PFC5) is perhaps the most commonly used in ADV because of its good combination of high vapor pressure, low solubility in blood, price, and availability. Other phase changing perfluorocarbons that have been explored are perfluorodichlorooctane [6] and perfluoro-15-crown-5-ether [7]. 

## 2. Physical Chemistry of Acoustic Droplet Vaporization

### 2.1. Thermodynamics of Vaporization

The first concepts pertaining to ADV are those of thermodynamic phase state (gas, liquid, and solid), phase change, and vapor pressure. Solids and liquids possess a vapor pressure, which is defined as the pressure of the specified gas in equilibrium with its own liquid or solid in a closed system at a specified temperature. The vapor pressure increases as the temperature of the condensed phase increases. Sometimes it is convenient to think of a vapor pressure as the “push” of the solid or liquid molecules trying to escape (the solid or liquid) to the gas phase. When the surrounding pressure is greater than the vapor pressure, the condensed phase remains in its condensed form (liquid or solid), although it may eventually dissipate as the material slowly dissolves and diffuses away into the aqueous phase. However, when the surrounding local pressure decreases below the vapor pressure, then the liquid molecules will quickly escape to form a gas phase (boil), or a solid will quickly sublime to a gas. The “normal boiling point” is the temperature at which the vapor pressure of a liquid is exactly 1 atm pressure; at this temperature the gas and liquid phases are in equilibrium, and a gas phase can form (depending on the volume of the closed system); above this temperature the liquid will transform (or boil) to a gas. Likewise, a gas of a single species will condense to a liquid when the local temperature decreases such that the liquid vapor pressure is below the surrounding gas pressure, or when the local gas pressure increases above the vapor pressure.

Acoustic droplet vaporization employs these concepts of vapor pressure, gas phase, and liquid phase, with the caveat that the change in local pressure causes the phase transformation, not necessarily any changes in temperature. When the local pressure drops below the liquid vapor pressure, the liquid can turn to gas. The acoustic aspect of ADV occurs because sound waves (which are pressure waves) are used to manipulate the local pressure of the liquid, thus controlling the drive to turn a liquid to gas, or gas to liquid.

### 2.2. Nucleation and Driving Forces

While it is true that a liquid whose vapor pressure is above the surrounding local pressure has a thermodynamic driving force to turn to gas, this transformation may not happen instantaneously for at least two reasons. First, heat needs to be transferred to the liquid because the change from liquid to gas required a certain amount of thermal energy; this is called the enthalpy (or heat) of vaporization. 

Second, a nucleation event is required to get the transformation started. It is possible and actually very common for liquids to remain in the liquid state even when above their boiling point (their liquid vapor pressure is above the local pressure) because a nucleation event must first occur to initiate the formation of the gas phase. A liquid above its boiling point is called “superheated.” The nucleation of a gas phase requires the formation of a nanoscopic cavity of gas (homogeneous nucleation), which in some cases continues to grow into a macroscopic gas phase (bubble). This process is random and stochastic, with an activation energy barrier that is related to the amount of superheating and the interfacial energy of the gas-liquid boundary [10]. The details are beyond the scope of this review, but the important result is that a liquid can remain superheated if it is very pure. The number of random events that create nucleation cavities in a superheated liquid is proportional to the volume of the liquid and the time length of observation, so a very small droplet has a much greater probability of remaining a superheated liquid for a longer time than has a large volume of liquid. Impurities, foreign surfaces, physical stresses, and higher amounts of superheating increase the probability that a gas bubble will nucleate within a superheated liquid. The probability of forming a nucleation cavity is also a strong function of the amount of superheating above the boiling point, in which superheating is often referred to as the “driving force” for nucleation.

In this review we define true superheating as the state when the vapor pressure of the liquid is above the vapor pressure of the surrounding material, such as when a large droplet (having negligible Laplace pressure) of liquid perfluoropentane in water at 30°C (having a vapor pressure of 1.02 atm) is surrounded by water at a pressure of 1 atm. Engineers also define superheating as any time a liquid is above its normal boiling point, such as pressurized water at 110°C (with a vapor pressure of 1.41 atm) confined in a pipe with a local pressure of 3 atm (304 kPa). However, we will call this situation “apparent superheating” because, although the liquid temperature is above its normal boiling point (at 1 atm), the liquid vapor pressure is still below the local pressure (3 atm), so it will never change to a gas no matter how long one waits.

A small liquid droplet that is immiscible in the surrounding liquid can experience this “apparent superheating” because of Laplace pressure, and thus will never change to a gas when confined inside the small droplet. Laplace pressure is the additional pressure imposed upon the interior fluid of a droplet because of the surface tension (or interfacial energy) between the two immiscible phases that compresses the liquid or gas inside the droplet. Although there are more sophisticated definitions and explanations of Laplace pressure [11], this qualitative description will suffice for this review. The magnitude of the Laplace pressure is given by Δ*P* = 2*γ*/*R*, where Δ*P* is the increase in pressure inside a spherical droplet of radius *R* compared to the local pressure of the surrounding fluid and *γ* is the interfacial energy. 

For example, consider a 1-micron-diameter droplet of PFC5 in water at 37°C and 1 atm pressure (local pressure of the surrounding water). The interfacial energy between water and PFC5 at this temperature is estimated to be 56 mN/m [12], and thus the Laplace pressure is calculated to be 224 kPa. This value plus the surrounding water pressure of 101 kPa produces an internal pressure in the droplet of 325 kPa. Even though the local temperature of 37°C produces a PFC5 vapor pressure of 132 kPa, the PFC5 will never turn to gas within this droplet because its vapor pressure remains lower than the local pressure inside the droplet. Although the droplet may slowly shrink due to diffusion of PFC5 into the surrounding water, this droplet will never boil (change to gas) even though it is (apparently) superheated above its normal boiling point of 29.2°C. The PFC5 liquid in this droplet would not turn to gas until it was heated above 66.2°C, so the smallness of the droplet creates an increased boiling point of the liquid. Good examples of apparent superheating and of increased boiling temperatures of perfluorocarbons are given by Sheeran et al. [13, 14]. (Note: vapor pressures and other thermodynamic properties for PFCs are taken from the DIPPR database [5]; properties of water are taken from steam tables [15].)

### 2.3. Ultrasound and Subpressurization

**Figure 1:**
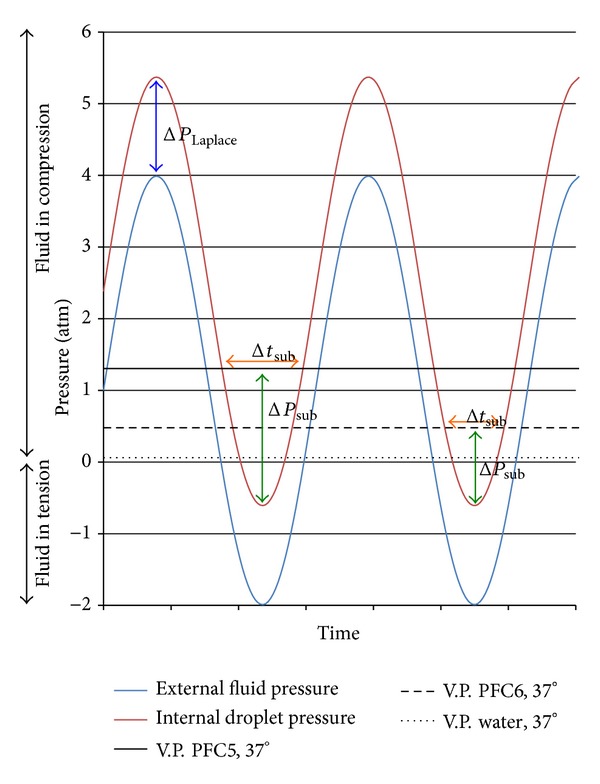
Plot of pressure in an ultrasonic wave. Upper sinusoidal line represents the pressure inside a PFC droplet of 1 *μ*m in diameter. Lower sinusoidal line represents the pressure of the surrounding fluid as the ultrasonic wave passes. The difference is the Laplace pressure. The vapor pressures of PFC5, PFC6, and water are indicated. The vertical arrows indicate the maximum subpressurization, and the horizontal arrows indicate the available subpressurization time.

Now, what does ultrasound have to do with all of this? Ultrasound is defined as pressure waves with a characteristic frequency greater than 20 kHz, the nominal upper threshold of hearing for humans. The wavelength of sound in water at room temperature is given by *λ* = *c*/*f*, where *c* is the phase velocity (speed of sound) and *f* is the frequency. For water at 37°C, *c* = 1,524 m/s, so wavelengths at 20 kHz, 1 MHz, and 5 MHz are 7.6 cm, 1.5 mm, and 305 *μ*m, respectively. All these lengths are much greater than the size of a 1 *μ*m PFC droplet, so we can consider that there are not significant pressure gradients through the volume of the droplet. The pressure of the fluid surrounding the droplet rises and decreases, and so does the pressure inside the droplet, although at a higher value due to the additional Laplace pressure, as Figure 1 shows. We note that Figure 1 indicates that the pressure can have a negative absolute value. This is possible because the strong cohesive forces in water (and presumably also in PFCs) allow the fluid to be placed in tension (negative pressure) without cohesive failure [16].

Referring to Figure 1 again, we show that during some sections of the acoustic pressure cycle, the internal pressure within the PFC droplet drops below the vapor pressure of the PFC and then increases again to values above the vapor pressure. During the short time window when the internal pressure is less than the vapor pressure (called subpressurization), there is a “driving force” for a gas phase to form. Experimental observations show that often a gas phase is not formed in some cases in which the subpressurization driving force is small (low acoustic amplitude) or the time window is short (high ultrasonic frequency). Thus the instant formation of a gas phase is not guaranteed, suggesting the requirement for a nucleation nidus or other nucleation event. Nucleation theory indicates that at small values of subpressurization in the absence of a heterogeneous nucleation event (particle nidus, shear or shock event, etc.) homogeneous nucleation will eventually occur, but it is a random or stochastic process. The probability of homogeneous nucleation of a growing gas bubble is proportional to the time window (at constant subpressurization) and increases exponentially with the magnitude of subpressurization. Thus ADV events will increase as the ultrasonic frequency decreases, as the number of cycles in a pulse increases, as the peak negative pressure of a wave increases in magnitude, and as the Laplace pressure decreases (due to lower interfacial energy or to larger droplet radius). Many of these postulates have been confirmed experimentally [17–19]. All of these factors should be considered in the analysis and optimization of ADV. 

Several authors have observed subpressurization of perfluorocarbon liquid droplets without gas formation [4, 9, 17–20]. This could be due to “apparent subpressurization,” analogous to apparent superheating, in which the Laplace pressure was sufficiently large that the vapor pressure at a temperature above the normal boiling point was still not greater than the local pressure inside the droplet. Or it could have been true subpressurization in the absence of a nucleation event. It is difficult to discern which of these occurred in the literature reports since the interfacial energy (and thus the Laplace pressure) was not always known or reported.

For example, several authors have reported that small PFC5 (b.p. 29.2°C) and PFC4 (b.p. −1.3°C) droplets are stable at 37°C [7, 17, 21–27]. But relatively few have also reported the droplet interfacial energy and size, from which the Laplace pressure can be calculated and true subpressurization can be calculated [15, 19].

### 2.4. Bubble Growth

High-speed photography of the nucleation of the gas phase shows that the bubble forms within the liquid PFC and not at the liquid PFC/water interface [23]. In some cases 2 bubbles nucleate within the droplet and may coalesce into 1 bubble [19].

**Figure 2:**
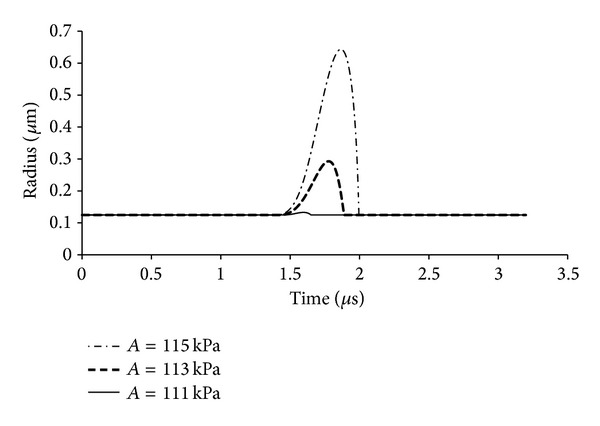
Plot of the radius of an expanding bubble as a function of time and acoustic amplitude. A 125 nm radius droplet of perfluorohexane in water was subjected to 500 kHz pressure waves with amplitudes of 111, 113, and 115 kPa. The temperature was 25°C, and the interfacial tension was 3.5 mN/m. The plot was adapted from [8].

**Figure 3:**
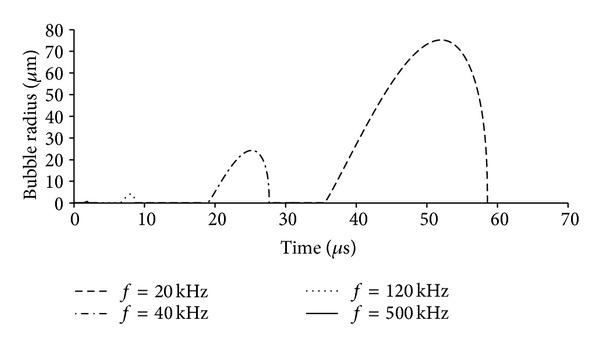
Plot of the radius of an expanding bubble as a function of time and acoustic frequency. A 125 nm radius droplet of perfluorohexane in water was subjected to a 500 kHz pressure wave with an amplitude of 110 kPa. The temperature was 25°C, and the interfacial tension was 3.5 mN/m. The plot was adapted from [8]. Each droplet expansion starts at a different time, because at lower frequencies, longer time is required before the pressure cycle drops low enough to cause the liquid to expand to gas.

Once the gas bubble is nucleated, it will continue to grow as long as the subpressurization exists and there is sufficient heat transfer to satisfy the required heat of formation of the gas phase. Heat transfer is usually not a limiting issue for small droplets of more than a few degrees of superheating [8]. In an acoustic field, the pressure eventually reverses, and the increasing internal pressure of the droplet eventually overtakes the vapor pressure; at this point the gas phase can condense back into liquid, and the liquid droplet is pressurized until the cycle repeats itself. The dynamics of such a system have been observed experimentally [17] and have been modeled for PFC5 and PFC6 droplets in water at 25 and 37°C [8]. As Figure 1 shows, the length of time of subpressurization is a function of the acoustic amplitude and frequency. Greater acoustic amplitude will start bubble growth sooner and have more total time for growth. Similarly, low frequency ultrasound provides a longer time window for growth. Figures 2 and 3 show how sensitive the bubble size is to the amplitude and frequency of the ultrasound as calculated from mathematical models. For example, increasing the acoustic amplitude from 111 kPa to 115 kPa increases the bubble size by more than a factor of 10 (see Figure 2). Decreasing the frequency from 500 kHz to 20 kHz increases the bubble size by more than a factor of 100 (see Figure 3).

After the insonation stops, the final state of the droplet may be a liquid, but there are some cases in which a gas phase may prevail. The first case may be a situation in which a condensed liquid droplet and expanded gas phase are both possible equilibrium states, and the gas phase persisted after cessation of insonation. For example, a 100 nm emulsion droplet of PFC5 coated with a layer phosphatidylcholine is predicted to have an interfacial energy of 3.5 mN/m [28] and thus a Laplace pressure of about 140,000 Pa. At 37°C, the PFC5 vapor pressure is about 132,000 Pa [5], which is less than the sum of the atmospheric pressure (101,000 Pa) and the Laplace pressure; this small droplet in the liquid state, although apparently superheated, is stable. However, if this size of liquid droplet was turned to PFC5 gas at 1 atm pressure, the diameter would be about 517 nm, and the Laplace pressure would be reduced to about 27,000 Pa (assuming the same interfacial energy). The internal pressure in the gas bubble would be about 128,000 Pa, slightly lower than the vapor pressure at 37°C (132,000 Pa); so this gas bubble would also be stable [29]. 

A more likely and perhaps ubiquitous experimental example is the case in which a noncondensable gas (nitrogen, oxygen, etc.) is dissolved in the liquid surrounding the droplet undergoing liquid-gas-liquid cycles during insonation. During the time that the gas phase is present, dissolved noncondensable gas (e.g., nitrogen) may diffuse to the gas-liquid interface and enter the expanding bubble of PFC gas. During contraction when the PFC condenses back to liquid during the high pressure phase of the acoustic cycle, the noncondensable gas will not condense along with the PFC and may not completely dissolve back into the surrounding liquid, leaving a very small bubble of noncondensable gas that easily nucleates the next cycle of PFC boiling, leading to an even larger bubble on the next cycle, and subsequently more diffusion of noncondensable gas into the bubble. At the end of several pressure cycles in the insonation pulse, a stable gas bubble may remain that is a mixture of PFC and noncondensable gas. This process has been observed and modeled [4, 23] and may explain several observations showing that gas bubbles following ADV are larger than would be expected given the amount of PFC in the initial liquid droplet [18, 24, 30, 31]. There are reports of other anomalous behaviors of very small PFC5 droplets forming gas bubbles much larger than expected, and this large size persists after the acoustic pulse has passed [24].

### 2.5. Thresholds for Bubble Formation

If we ignore the required nucleation of a gas phase, the peak negative pressure threshold for ADV can be easily calculated from the vapor pressure, Laplace pressure, and local hydrostatic pressure. Experimental observation of thresholds indicates that ADV does not readily occur until much greater ultrasonic amplitudes are applied. For example, Kripfgans et al. measured ADV thresholds for 8 *μ*m PFC5 droplets in water at 23°C with a reported interfacial tension of 33.8 mN/m [19]. Assuming a local pressure of 101 kPa, the Laplace pressure plus local pressure in the droplet is calculated to be 117.9 kPa, and the vapor pressure at 23°C is 79.4 kPa. This difference is only 38.5 kPa, so any acoustic wave larger than this would drop the internal pressure below the vapor pressure. However, the experimentally observed threshold of 1.7 MPa at 3 MHz is nearly 2 orders of magnitude greater than the calculated theoretical minimum of 38.5 kPa. 

In other experiments, Sheeran et al. made 200–300 nm perfluorobutane (PFC4) droplets in water with an estimated interfacial energy of 30 mN/m [17]. The droplet internal pressure of 0.58 MPa, less the 0.28 MPa vapor pressure of PFC4 at 25°C, is only 0.30 MPa, and yet the observed ADV threshold was 1.45 MPa at 1 MHz frequency.

Giesecke and Hynynen made PFC5 droplets stabilized with albumin but did not report an interfacial tension; therefore the Laplace pressure cannot be estimated [18]. However, they found that 2 *μ*m diameter droplets formed vapor without insonation at 72°C. At 37°C with insonation, gas formation was observed at 0.65 MPa at 0.74 MHz and at 1.05 MPa at 1.1 MHz. Other similar observations regarding thresholds have been made [32, 33]. Interestingly, the threshold at high frequency appears to be dependent upon the duration of the insonating pulse [34], again hinting that bubble nucleation is not instantaneous.

## 3. Acoustic Droplet Vaporization in Clinical Nanomedicine

The majority of clinical research using ultrasound for vascular imaging has employed the use of microbubbles (MBs) as contrast agents to enhance the acoustic signal from the blood. MBs are gas-in-liquid bubbles most often stabilized with albumin, galactose, lipid, or polymers [35]. The average diameters of the MBs are generally around 2.5 *μ*m and can range from 1 to 10 *μ*m. The MBs resonate in an ultrasonic field, rapidly contracting and expanding in response to the pressure changes of the sound waves [36]. 

While micron-sized gas-phase contrast agents are easily introduced into blood, their large size precludes their entry into the extravascular space [37] and also promotes more rapid clearance. Larger particles are taken up more readily by the cells of the reticuloendothelial system (RES) [38–40]. Therefore, emulsions containing submicron and nanometer-sized perfluorocarbon (PFC) droplets that can change to gas are being studied in diagnostic and therapeutic applications of ultrasound. PFCs that form gas are also studied for ultrasonic molecular imaging, the targeted delivery of some therapeutic agents, and in phase aberration correction. Recently, the use of liquid-phase PFC droplets that remain in the liquid state has been explored as a contrast agent [41]; however, this application is not reviewed herein.

The following review presents four applications of ADV in clinical settings and discusses their future possibilities.

### 3.1. ADV in Vascular Imaging

 In general, the aim of ultrasound contrast agents is to selectively increase the strength of the back-scattered signal that is returned to the detecting transducer. The first clinical application of phase changing emulsions as an ultrasound contrast agent appears to be in 1995 with a product called EchoGen made by Sonus Pharmaceuticals (Bothell, WA). EchoGen was a suspension of PFC5 liquid droplets in water, stabilized by an albumin layer. The reported droplet size was 0.3 *μ*m in diameter, and the bubbles produced were reported to be from 1 to 10 *μ*m, with an average diameter of 6 to 8 *μ*m [42]. Since expansion at 37° produces only a 5-fold expansion in radius from liquid to gas, these large bubble sizes suggest that after phase transformation the smaller bubbles coalesced together and/or absorbed dissolved gas from the surrounding liquid. Although the authors of these early papers supposed that the bubbles were produced by thermal expansion of the liquid to gas, we now know that the droplets in EchoGen are fairly stable at 37°, and most, if not all, of the bubbles were generated by the excitation by the applied ultrasonic imaging pulses. Thus these are examples of acoustic droplet vaporization in its earliest application.

EchoGen was first reported in preclinical application to image the canine renal cortex, providing contrast between the cortex and medulla. It had a half-life of 2 to 3 minutes with an intravenous dose of 0.25 to 0.45 mL/kg [1]. As mentioned, in this and other early papers, bubble formation was attributed to the droplets “undergoing a phase transition to gas above 30°C.” There was no comparison to other conventional contrast agents of that time, so the comparison and advantages to imaging with Albunex were not reported.

In 1996 the first images of color Doppler analysis with EchoGen in human kidneys appeared, using a lower dose than was used in animals (0.05 mL/kg) [31]. By 1998, EchoGen and related PFC5 phase-shift agents, SonoGen, and QW7437 (Sonus Pharmaceuticals) were being used for myocardial opacification in clinical trials [43]. EchoGen was also used to image the prostates of 15 patients using 7-MHz color Doppler linear array transrectal transducers [44]. After injection of 0.05 mL/kg of EchoGen, the entire prostate was examined to study the blood flow in the gland. The combination of the color Doppler sonography and EchoGen provided sufficient contrast in a number of vessels that could not be identified otherwise. No side effects were observed.

Many studies were made on left ventricle opacification; since the PFC5 liquid droplets were much smaller than gas bubbles, they could traverse the lungs and provide contrast by acoustic droplet vaporization in the left heart better than the then-FDA-approved contrast agent Albunex [42, 45, 46]. QW7437 was formulated with a negative surface charge so that it would not adhere to the vascular endothelium [47]. It appeared to deposit in the myocardium and provided myocardial contrast even after the ventricles had been cleared of contrast agent.

The pharmacokinetics of the PFC5 droplets (EchoGen) in human volunteers was investigated as part of the safety evaluation (0.01 to 0.1 mL/kg), showing that the PFC5 clearance of about 30 mL/min/kg was by exhalation [48]. Although adverse effects in humans have not been reported (at therapeutic doses), repeated administrations of high doses (0.5 mL/kg) in dogs produced evidence of accumulation in their lungs and eventual hemodynamic collapse [49]. An alternative method of activating the EchoGen to bubbles was published in 1998 [50]. The physician would pull back the plunger of a 20 mL syringe for a few seconds and then release it, generating a loud popping sound, and then he would inject the contrast agent. This low-pressure activation produced adequate contrast to image the liver and kidneys. It also provided some transpulmonary opacification of the left heart, again suggesting that the microbubbles were cleared to a lesser extent than conventional contrast agents used at that time [42, 47]. Although this application was not strictly “acoustic droplet vaporization,” it was a novel application of EchoGen that took advantage of the high vapor pressure of PFC5.

The range of tissues that could be imaged with PFC5 was expanded to basal cerebral arteries in 1999 [51]. As mentioned, we posit that the small size of the PFC5 emulsions and microbubbles produces slower clearance and thus retains sufficiently high concentration to allow imaging where none had previously been done. This study revealed the sensitivity that could be achieved with acoustic droplet vaporization of PFC5 emulsion droplets in transcranial imaging.

Interestingly, published papers of clinical applications of EchoGen, SonoGen, QW7437, and perflenapent were absent after 2003. EchoGen was not approved by the FDA, and apparent interest and funding vanished. It also had competition from Definity, a microbubble contrast agent containing perfluoropropane, which was introduced in 1999 [52] and had reduced clearance by the RES system by virtue of its polyethylene glycol (PEG) coating. However, the use of PFC5 emulsion droplets in other applications started to increase, including the use in vascular occlusion, molecular imaging, and therapeutic delivery.

### 3.2. ADV in Vascular Occlusion

Another well-studied application of ADV is embolotherapy. Successful application of embolotherapy requires an understanding of the disease to be treated, the distinctive features of the circulation to be embolized, and the embolic material used for the occlusion [53]. Embolotherapy must be carefully done because many arterial emboli could create infarcts in the heart or brain or travel to distant vascular bed where they could cause unwanted arterial occlusion, ischemia, and potentially infarction [54]. However, one method of treating tumors or other malformations is to occlude the blood flow to the tissue with gas bubbles, which can effectively shrink the tumor. As an added advantage, embolotherapy using ADV also enables simultaneous imaging and therapy in cancer treatments.

In practice, embolotherapeutic occlusion is done by focusing ultrasound on arterioles feeding a tumor. As the PFC droplets flow through the targeted vasculature, the droplets expand to gas, which often occludes the further flow of blood and produces ischemic damage to the downstream tissues [4]. PFC droplets that are not activated to gas are too small to cause embolism downstream. Currently, ADV with micron-sized PFC droplets is applied in preclinical use for staging and prognosis of hepatocellular and renal carcinoma [55, 56]. In other types of cancer, ADV is a well-accepted concept; yet current ADV techniques that include using perfluorocarbon droplets as contrast agents, such as perfluoropentane (PFC5), have not been widely validated. Due to the low solubility and diffusivity of PFC gases in water, bubbles can remain stable in an aqueous solution much longer than air bubbles of the same size [57]. These properties endow the PFCs droplets with desirable properties for applications in clinical occlusion.

Samuel et al. prepared 2 *μ*m (mean diameter) albumin-encapsulated PFC5 droplets (1 × 10^8^ droplets/mL) in normal saline. After injection of this solution via the carotid artery, ultrasonically activated bubbles (3.5 MHz, 6 MPa, 3.7 *μ*s pulse length, and 10 Hz pulse repetition frequency) occluded the 125 *μ*m (average diameter) arterioles and the 4–7 *μ*m capillary beds in Sprague-Dawley rats [58]. To obtain microscopic evidence of this occlusion, intravital microscopy was used to image the droplet bubbles that caused occlusion. In addition to occlusion, the different images of erythrocyte extravasation indicate that insonation of the PFC5 droplets produced bubble oscillations and probably inertial cavitation, which resulted in the rupture of arterioles and/or capillaries.

Other studies by the same group showed that ADV following injection of 2 *μ*m PFC5 droplets caused occlusion in canine kidneys [59, 60]. Image-based hyperechogenicity showed that the tissue perfusion was changed after injection and insonation at 3.5 MHz, 7.4 MPa at the focal point, with a 1 kHz pulse repetition frequency (0.25% duty cycle). Successful ADV demonstrated the potential of this technique to occlude flow [60]. 

### 3.3. ADV with Molecular Recognition in Cancer Detection

Early detection of cancer remains one of the most desirable goals for tumor imaging, particularly for identification of early primary tumors and of metastatic spread. Two techniques can be used. First, the tumor vasculature is often malformed and may be detectable by imaging [61]. Second, ligands that specifically bind to the tumor can be attached to the imaging agent to identify tissues of cancerous phenotype. Therefore, there is interest in the potential application of ADV to identify molecular target expression in primary and metastatic cancers. In theory, labeled nanodroplets can penetrate the endothelial barrier and attach to cells expressing surface features indicative of cancerous phenotype [62]. 

With the growth in ADV-based imaging of cancer as demonstrated in preclinical settings, there is a strong argument for efforts to apply ADV to clinical monitoring of cancer therapies. Early work has demonstrated in animal models that ADV-based angiography can provide sensitive feedback on the effect of ultrasonic therapy in models of pancreatic cancer, breast cancer, kidney function, and so forth [7, 21]. Matsuura et al. used quantum dots, loaded into PFC5 droplets, to show that the droplets could be converted to gas and imaged at 18 MHz with the application of 4.7 MPa acoustic pressure and 32 cycle bursts of ultrasound in hepatoma in mice by ADV [55]. Additional approaches have used antibodies and peptides to target imaging agents for treatment of emerging glioma. An example of this is the use of nanoparticle labeled antivascular endothelial growth factor receptor (EGFR) antibodies for *in vitro* and *in vivo* magnetic resonance molecular imaging. This approach successfully visualized and differentiated C6 glioma tumor types based on their EGFR expression [25]. Intravenous application of labeled anti-EGFR in this same study resulted in a quick delivery to the tumor without the necessity to clear the tissue from adherent tissue or mucus. In this preclinical study, the particles collected in the rat brain, but insonation was never applied to expand the PFC5 droplets to gas to release the drug or permeabilize the blood-brain barrier. A similar construct employing ADV and featuring aptamers for targeting to deliver Doxorubicin has also been described but not employed yet in clinical studies [63]. 

### 3.4. ADV in Therapeutic Delivery

Implementation of ADV in the delivery of drugs, plasmids, and other therapeutic agents can be divided into two general categories. The first is the use of ultrasonic ADV to produce gas bubbles on demand at the site of interest and then employ the newly formed gas bubbles as cavitating bodies that accomplish drug delivery by the same mechanisms that gas bubbles normally employ. These include cavitational disruption of drug carriers and sonoporation of cell membranes, the latter leading to increased cell permeability. The second general application of ADV is to use the PFC droplet as a contrast agent, either in the liquid or gas form, to visualize and confirm the location of the desired delivery; then when or if the location is correct, the liquid or gas is subjected to higher intensity ultrasound to generate intense cavitational events that can disrupt carriers or cell membranes. This combination of diagnostics followed by therapeutics (with the same construct) is called theranostics. This section will present clinical (or near clinical) examples of these two general approaches. 

A search in September of 2013 revealed that there were yet no published reports of the application of ADV for therapeutic delivery in human medicine. However, there were several reports of ADV in mice, indicating that this technology was approaching, but had not yet arrived, in clinical medicine. 

#### 3.4.1. Drug Delivery Using ADV

While there are no publications of ADV for clinical drug delivery, there are some articles that describe the use of ADV for drug delivery to tumors in mice. These are from the laboratory of Dr. Natalya Rapoport of the University of Utah. The first study employed a formulation of doxorubicin-containing block copolymer micelles (poly lactic acid-polyethylene glycol, PLA-PEG) mixed with perfluoropentane [20, 64]. The carriers were formed by sonicating (at 20 kHz) a mixture of drug-loaded micelles and liquid PFC5. The resulting formulation had PFC5 nanosized droplets that they claimed were stabilized by some of the block copolymer and by some of the whole micelles, with the drug distributed in both the micelles and the nanodroplet surface. By adjusting the ratio of PFC5 to micellar suspension, they achieved a mixture of droplets and micelles that was injected into *nu/nu* mice bearing breast and ovarian tumors. In their first two papers they hypothesize that the droplets may have been transformed to “drug-loaded nanobubbles” by thermal activation before they extravasated in mouse tumors. However they did not measure what fraction of PFC5 droplets may have been thermally activated. They also hypothesized that both the micelles and nanobubbles extravasated into the tumor. The presence of the nanobubbles allowed imaging of the tumor at 14 MHz. 

To execute the drug release, the tumors were insonated at 3 MHz and 2 W/cm^2^ at a 20% duty cycle for 150 sec, resulting in cavitation of the thermally activated bubbles that released the drug they carried and also induced release from the nearby micelles. Following 4 treatments in 2 weeks, the tumors did not continue to grow at the same rate as control tumors (no carrier, no insonation) and as tumors that received the carrier but without insonation. In some cases the tumors grew again after several days, indicating that tumor therapy was transient. While the authors did not mention acoustic bubble vaporization by name, ADV probably did occur with liquid PFC5 nanodroplets that were small enough that the Laplace pressure prevented their thermal activation. Thus these studies are the first known publications of ADV for drug delivery in an animal model. The study also demonstrated the potential of theranostics in which the PFC5 nanodroplets functioned both to provide ultrasound contrast and drug delivery.

**Figure 4:**
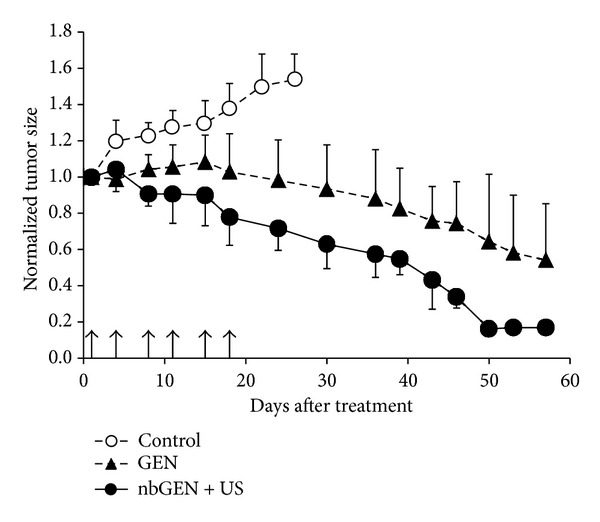
Breast tumor growth in mice for control tumor (open circles), tumors treated with a micellar PTX formulation (filled triangles), and nanodroplet PTX formulation combined with ultrasound (filled circles). Mean values plus/minus standard error are presented (*N* = 3). Arrows indicate days of treatment. Adapted by permission from reference [9].

The same research group did a later study that was similar to the first but which employed paclitaxel (Ptx) instead of doxorubicin, and the insonation parameters were slightly different [9]. Again micelles of Ptx in block copolymers were formed and mixed with a quantity of PFC5, followed by sonication at 20 kHz to form a mixture of micelles and 700 nm (peak average) nanodroplets stabilized by some of the polymer. This study enrolled mice bearing breast, ovarian, and pancreatic tumors. After intravenous injection, the tumors were visualized at 14 MHz and treated with 1 MHz insonation at 3.4 W/cm^2^ for 1 min. This treatment was given 4 times in 2 weeks (ovarian cancer model), 6 times in 3 weeks (breast cancer model), or 8 times in 6 weeks (pancreatic cancer model). In all cases, the tumors receiving the formulation with insonation grew at a slower rate or regressed more than other controls.  Figure 4 shows an example of tumor regression in the mouse model of breast cancer.

In this second study, the authors discussed acoustic droplet vaporization extensively, including the role of Laplace pressure and temperature. They attributed the positive results to liquid-to-gas transition in the tumor. They also discussed that droplets could be converted to gas by thermal processing and shearing in a syringe needle, in addition to acoustic activation. In a final interesting note, they observed that tumors treated by insonation of their formulation had less evidence of metastatic spread, which argues against the notion that ultrasonic cavitation in tumors can promote metastasis [65].

In a similar study to that mentioned first, a group in Beijing made a slight variation to the constructs of the Rapoport group. They used PLGA-PEG (poly lactic glycolic acid—PEG) instead of PLA-PEG to form doxorubicin-containing micelles; then they added PFC5 and sonicated to form stable droplets of less than 200 nm [66]. They claim that these were thermally activated to form gas bubbles by injection into mice at 37°C. The mice in their experiments hosted H22 tumors (mouse hepatocarcinoma). Again, the nanodroplet formulation combined with 40 kHz ultrasound at 0.7 W/cm^2^ effectively suppressed tumor growth for 6 days, while the tumor continued to grow in controls without ultrasonic activation and in controls with neither ultrasound nor formulation. The report makes no mention of droplet vaporization, but probably ADV occurred since their droplet size was so small that Laplace pressure probably retained some of the droplets in a liquid state until ultrasonically activated. 

#### 3.4.2. Mechanisms of Drug Delivery

Unfortunately, none of the reports described above provide much accompanying evidence of mechanism *in vivo*. The Rapoport group published several papers of *in vitro* observations including cavitation thresholds [7, 67] and microscopic observations of bubble formation [67–69]. Both the Rapoport and the Du groups propose various scenarios and hypotheses that claim to be consistent with their *in vitro* and *in vivo* observations, but which are difficult to prove *in vivo*; yet these hypotheses must be substantiated before clinical application can commence. 

For example, the group from Utah proposed that their nanobubbles enter the tumor by extravasation [20, 64]. The optimal size for extravasation is generally considered to be 100 to 300 nm [70], but particles as large as 750 nm may be extravasated in some tumors [37, 71]. In Rapoport's experiments with Dox-loaded constructs, a formulation of 0.5% polymer and 1% PFC5 was reported to form a bimodal distribution of droplets with peak sizes of 250 and 1328 nm. In the mouse experiments, a formulation of 0.5% polymer and 2% PFC5 was employed, but its size was not reported, so the PFC5 droplets may have been somewhat larger in those experiments. The smaller droplets may have extravasated if they were not thermally activated to gas droplets before insonation. Transformation of a 250 nm droplet to gas would result in a 1250 nm bubble, too large to extravasate. The observation that the combination of ultrasound and formulation was effective in retarding tumor growth suggests that nonactivated droplets did extravasate and became acoustically activated or that gas bubble formation and cavitation may have occurred in the capillaries in the tumor, leading perhaps to capillary disruption or at least increased capillary permeability to the drug or drug carriers (micelles or other droplets).

The Dox-loaded nanodroplets of the Chinese study were on the order of 160 nm in diameter, so they could have extravasated before acoustic activation [66]. However, if they were thermally activated to gas bubbles (as the authors claim), they may have been too large (~800 nm) to extravasate.

In the study of Ptx-loaded nanodroplets, the nanodroplets had a peak diameter of 700 nm [9]. These may have extravasated, although they are larger than the optimal size.

To comment on observations, in pursuing future studies it will be critical to know both the phase state (liquid or gas) and the size of particles in animal studies so that hypotheses can be carefully formulated and tested. It would also be very useful to collect insonated and noninsonated tumors with the goal of assessing if accumulation of carriers is occurring (via extravasation) and perhaps perform microscopy work to validate the extravasation hypothesis. Another piece of information, perhaps more difficult to collect, is what effect a cavitating PFC5 bubble has upon local tissue within a tumor.

### 3.5. Other Applications of ADV

There are two other applications of ADV that have great potential for future clinical use. These are the use of ADV to form gas cavities through which much thermal energy can be deposited and the use of ADV in aberration correction. To our knowledge, neither is yet approaching clinical trials.

One intriguing application of ADV is its use as a nucleation agent for bubble-enhanced tumor ablation by high-intensity focused ultrasound (HIFU) [72]. In clinical practice, HIFU has been applied to treat solid malignant tumors, including the liver, prostate, breast, bladder, kidney, and soft-tissue sarcoma [73]. Absorption of the ultrasound energy in the focal area can produce localized temperature elevations and generate tissue necrosis without damaging the surrounding tissues [74]. Therefore, HIFU ablation provides a noninvasive modality with precise targeting of tissues for cancer treatment. Transformation of ultrasonic pressure waves to thermal energy is much more efficient in the presence of bubbles that oscillate and collapse, producing localized viscous heating. Recently, HIFU has been used with ADV of PFC5 nanoemulsions to enhance the heating produced by focused ultrasound *in vitro* and *in vivo* studies [22, 72, 75]. Their work has demonstrated that the nanoemulsions can be an effective nucleating agent for acoustic cavitation and can be employed to enhance HIFU-mediated heating locally. ADV may provide a means of increasing localized thermal ablation for cancer therapy that hopefully will soon be demonstrated in a preclinical setting.

Aberration correction is a mathematical technique applied to ultrasonic imaging data that corrects for the distortions that occur as ultrasound travels through various tissues [76]. Aberration is particularly annoying during imaging within the skull because of the various thicknesses and densities of cranial bone. One method to make the correction is a point-target technique that relies on sparsely distributed fixed points in space, imaged from various angles, from which aberration corrections are calculated [77]. Gas bubbles are a good source of point reflections [78], but introducing bubbles into the brain could potentially be problematic if they coalesce and occlude capillaries, and intra-cranial injection of bubbles is challenging. As before, the intravenous injection of small PFC droplets with slow clearance rates provides distributed points of gas in the brain when activated by transcranial ultrasound. ADV of PFC5 droplets has been proposed [79] and then applied in *ex vivo* skull models [76] and tissue mimicking gels [80]. While not yet in the clinic, ADV for aberration correction is a very promising strategy that could be developed for very controlled HIFU treatment of cancer or for precise drug delivery to the brain.

## 4. Clinical Potential and Application of Acoustic Droplet Vaporization

Applications of ADV in cardiac and vascular imaging first commenced nearly 2 decades ago, and then diminished within 10 years. While currently obsolete, this wave of using PFC5 droplets and microbubbles for imaging occurred because the droplets and perhaps microbubbles were sufficiently small that their clearance was slow and provided sustained ultrasound contrast for imaging the left heart and arterial circulation. Such imaging prior to that time could not be done at that time without intra-arterial injection, which is problematic both then and now. However, the ADV contrast agents were never approved by the FDA following clinical trials. In the late 1990s, the contrast agent Definity appeared. This small microbubble of perfluoropropane apparently found better clinical acceptance than EchoGen and its sister products. In our opinion, the use of ADV for standard clinical imaging of cardiovascular organs and systems will probably not experience any resurgence. Better contrast agents have come along, and hopefully even better contrast agents will arrive in the future. However, the brief use of ADV for vascular imaging set the stage for current and future applications in other areas, including occlusion, drug delivery, molecular imaging, and aberration correction. 

 To our knowledge, ADV for vascular occlusion has not yet been used clinically, although it has been used in animals [55, 58]. This application of ADV has significant clinical potential for several reasons. First, the perfluorocarbon droplets can be intravenously injected at a convenient site, can remain in the circulatory system, and then can be activated to form gas bubbles only at the site of insonation. Second, the occlusive bodies (gas bubbles) are not permanent and do not need to be retrieved at a later time. They will eventually dissolve away. Thus there is no concern for the retrieval of or the permanent residence of metal, ceramic, or polymeric materials in the tissues. While there will be competition in the clinic from other modes of vascular occlusion, we foresee that there is great potential here for clinical application in vital organ tissues (brain, liver, eye, etc.) in which revascularization after therapeutic healing is desired. One of the challenges is to create droplets that have stealth character and yet when activated to gas bubbles will easily coalesce into bubbles sufficiently large to occlude arterioles and capillaries. Stealth character is usually endowed by incorporating polyethyleneglycol (PEG) chains in the surfactants that stabilize the vesicles [81]. However, the presence of PEG chains may cause bubbles to repel each other and moderate the coalescence. This optimal balance needs to be addressed.

Clinical application of ADV in molecular targeting and therapeutic delivery of drugs and genes is probable but still requires much work. Preclinical animal models (mice) have shown potential. Both applications require very specific molecular targeting to attach the PFC nanodroplets to the correct tissues, although some therapeutic delivery could be done via passive targeting [82]. These applications of ADV in clinical medicine may be delayed until very specific targeting is developed further. The combination of imaging with drug delivery will be very powerful in clinical medicine. However, this same combination may slow the approval by regulatory agencies that currently do not have infrastructure for approval of combined devices, such as a combined imaging agent and a therapeutic drug [83]. This regulatory obstacle may temper the enthusiasm of pharmaceutical companies to pursue development, given the expense and risk of clinical trials.

While ADV for nucleation of bubbles in HIFU therapy may have clinical application, its use is probably years away for critical tissues such as the brain and vital organs. Similarly, we foresee that ADV for aberration correction may take some time, given that other correction algorithms are also competing for clinical attention.

## 5. Remaining Critical Issues

As with all therapeutic agents and procedures, thorough research must be done to ensure the safety and efficacy of the therapy. These concerns should guide the future directions of research in therapeutic applications of ADV.

From a medical point of view, issues in safety of targeted agents and safety of contrast agents are fundamentally important. This includes chemical safety (nontoxicity) and physical safety, the main concern of which is premature expansion leading to gas bubble occlusion of capillaries. While the perfluorocarbons of interest are deeded nontoxic, the surfactants that stabilize the droplets must also be considered. Natural phospholipids, polysaccharides, and human proteins may be the best stabilizing agents to use. Stabilization by synthetic polymers will remain suspect until nontoxicity is proven [84].

More data on the kinetics of phase transformation under dynamic shear stresses are needed to ensure to the medical community that premature and nontargeted expansion is a rarity. More measurements need to be done to establish the acoustic thresholds for gas expansion as a function of acoustic parameters (frequency, amplitude, pulse length, etc.) and the characteristics of the droplets (chemical composition, size, stabilizing surfactants, temperature, etc.). The medical community needs a reliable and controllable off/on switch to engender confidence that gas bubble will be formed only when and where they are desired. Uncontrolled formation of microbubbles may cause side effects observed in experimental studies, including hemolysis and endothelial damage [85, 86].

A safety issue (which very few if any studies have mentioned) is cavitation after formation of a gas bubble. If a bubble is formed at the beginning of an acoustic pulse, what possible damage may occur during the remainder of the pulse, caused by the oscillation and collapse of the cavitating bubble? In some cases, such as drug delivery, cavitation may be a desired by-product that may enhance drug delivery. In HIFU, strong cavitation is the desired effect. However, for molecular targeting, occlusion, imaging, and aberration correction, cavitation may produce unwanted tissue damage. In such cases it will be essential to know what combination of intensity and pulse length is necessary to form the gas bubble with minimal subsequent damage by cavitation.

Another safety issue is the development of sensitivity or allergic reactions to the ADV constructs. We have not found any reports of sensitivities to small amounts of perfluorocarbons; this needs to be studied in more depth. Also, attachment of proteins to nanodroplets as targeting ligands may lead to allergies to those proteins. 

As a foreign body, microbubbles may be cleared by the phagocytic cells of the reticuloendothelial system [87]. From 5 to 10 minutes after injection, the EchoGen (PFC5 gas) is exhaled via lungs, while the components of the shell are metabolized or filtered by the kidney and eliminated by the liver [88]. The microbubble sizes were 2–5 *μ*m in diameter and circulated in the body. Nonactivated PFC droplets are much smaller and may not be cleared by the same mechanisms as bubbles. More research is needed to estimate the residence times and clearance rates.

From a science and engineering viewpoint, the effectiveness of the nanodroplet is almost as important as safety. There are a number of issues regarding efficacy that still need to be addressed. These include very specific binding for molecular targeting and drug delivery, the size and design of stealth polymers in masking the constructs from the RES system, and the appropriate size for passive accumulation via extravasation. With respect to feasible commercialization, one must always consider the ease of manufacturing and the shelf life and storage of the product.

## Figures and Tables

**Table 1:** Properties of selected perfluorocarbons [5].

Common name	Chemical formula	Normal boiling point (°C)	Vapor pressure at 37°C (kPa)	Expansion ratio*
Perfluorobutane	C_4_F_10_	−1.3	387.98	5.37
Perfluoropentane	C_5_F_12_	29.2	135.05	5.17
Perfluorohexane	C_6_F_14_	57.1	48.09	4.97

*Diameter expansion ratio calculated assuming no Laplace pressure compressing the final gas bubble, internal bubble pressure at 1 atm, and temperature of 37°C.
